# Multicentricity in the thyroid differentiated carcinoma

**DOI:** 10.1016/S1808-8694(15)30838-7

**Published:** 2015-10-18

**Authors:** José Francisco Salles Chagas, José Luís Braga de Aquino, Maria Beatriz Nogueira Pascoal, Adriana Soave Teixeira, Márcia Maria Nunes Ferro, Mariana Cristina Ortiz Gambaro, Rogério Aparecido Dedivitis

**Affiliations:** 1 PhD in Medicine - Graduate Program of Otolaryngology/head and neck surgery - Universidade Federal de São Paulo - Escola Paulista de Medicina, Professor of Graduate Programs in Surgery at the Faculdade de Medicina da Pontifícia Universidade Católica de Campinas. Head of the Head and Neck Surgery Service - Hospital e Maternidade Celso Pierro, da Pontifícia Universidade Católica de Campinas.; 2 PhD in Medicine - Universidade Estadual de Campinas, Professor of Surgery - Faculdade de Medicina da Pontifícia Universidade Católica de Campinas. Surgeon at the Head and Neck Surgery Service - Hospital e Maternidade Celso Pierro, da Pontifícia Universidade Católica de Campinas.; 3 PhD Student - Universidade Estadual de São Paulo, Surgeon - Head and Neck Surgery - Hospital e Maternidade Celso Pierro, da Pontifícia Universidade Católica de Campinas.; 4 Medical Student - Faculdade de Medicina da Pontifícia Universidade Católica de Campinas.; 5 Medical Student - Faculdade de Medicina da Pontifícia Universidade Católica de Campinas.; 6 Medical Student - Faculdade de Medicina da Pontifícia Universidade Católica de Campinas.; 7 PhD in Medicine - Graduate Program in Otolaryngology and Head and Neck Surgery UNIFESP - Escola Paulista de Medicina (MD). Serviço de Cirurgia de Cabeça e Pescoço do Hospital e Maternidade Celso Pierro da Pontifícia Universidade Católica de Campinas.

**Keywords:** thyroid neoplasms, reoperation, thyroidectomy, thyroid gland

## Abstract

The treatment of choice for the well differentiated thyroid carcinoma has always been controversial. **Aim:** to analyze tumor invasion of the thyroid gland’s contralateral lobe in cases of differentiated carcinoma, correlating risk/benefit with the complications of a second surgical approach. **Materials and methods:** Retrospective study, from 1998 to 2006, of 27 patients undergoing less than total thyroidectomy: lobectomy (21), subtotal thyroidectomy (5) or isthmusectomy (1). Gender, age, type of surgery, complications, histopathological analysis and invasion of the contralateral lobe were analyzed. Patients’ ages varied from 17 to 89; the most frequent histopathological pattern was the classical papillary carcinoma (18 cases), followed by follicular carcinoma (6); the follicular variant of the papillary carcinoma (2) and the Hürthle cell carcinoma (1). Twenty-one patients underwent full thyroidectomies, from 15 to 30 days after the first intervention. **Results:** the contralateral lobe analysis was negative for carcinoma in 16 (76.5%) and positive in the other 5 (23.8%) patients. The complications observed were temporary dysphonia (3 cases) and hypoparathyroidism (2 cases, one permanent). **Conclusions:** total thyroidectomy is important in the treatment of differentiated thyroid carcinomas, because there is a high contralateral spread rate (23.8%). It is a procedure without mortality, which bears few complications.

## INTRODUCTION

Surgical treatment of choice in thyroid differentiated carcinoma has always been a reason for controversy. Some authors advocate unilateral lobectomy and isthmectomy, since there is no difference in survival, when compared with total thyroidectomy, and it also reduces postoperative morbidity. Others have shown the advantages of subtotal thyroidectomy in the treatment of well differentiated carcinomas (removal of the entire affected side, isthmus and part of the contralateral lobe), because it would reduce the rate of local recurrence when compared to the patients submitted to a unilateral procedure. There are still authors who favor total thyroidectomy, because of the high percentage of contralateral lobe involvement and because it is a procedure with null mortality and a low incidence of complications^1^.

The finding of a bilateral malignant lesion in clinically unilateral thyroid tumors grows in importance when we consider clinical and epidemiological stand point, as well as surgical and anatomopathological aspects. Such lesion or lesions located in the contralateral lobe and not diagnosed in the preoperative period (by semiotechnique and/or image exams) can be seen during the operation by the surgeon (through palpation), by the pathologist (during the frozen section intraoperative histopathology exam) or, as it happens most of the times, only during the final pathology exam, with the specimen already fixed in parafin^2^.

In the national literature, the data regarding the remaining thyroid tissue removal by completion thyroidectomy in the second operation is limited, and there is no more specific analysis in this aspect. Moreover, most publications on this topic present extremely variable results, about the bilateral prevalence of malignancy from twenty all the way to eighty-eight percent^2^.

The goal of the present study was to analyze the pathology results of thyroidectomy completion in a single series of patients with unilateral thyroid differentiated carcinoma, and assess the potential complications that could occur from the second procedure.

## MATERIALS AND METHODS

From 1998 to 2006, 36 patients with a diagnosis of thyroid carcinoma were operated. The study was approved by the Ethics Committee of the institution where it was carried out under protocol number 0282.0.147.000-07. Total thyroidectomy was performed as first intervention in nine patients (25%). In 21 patients (58.3%), the first intervention was lobectomy with isthmectomy, since these patients had a solid nodule at the preoperative neck ultrasound. Subtotal thyroidectomy was carried out in five patients (13.9%) because they had multinodular goiter in both thyroid lobes and the remaining patient was submitted to isthmectomy (2.8%), because this patient had a single nodule in the thyroid isthmus.

All the patients were assessed in the preoperative by means of ultrasound, fine needle aspiration of the nodule and lab tests to evaluate thyroid function.

Regarding the epidemiological profile of the patients initially submitted to unilateral surgery or to subtotal thyroidectomy, 23 patients were females (85.2%) and four were males (14.8%). Age varied between 17 and 89 years, with a mean value of 45 years.

The pathology analysis of the surgical specimens revealed the classic variant papillifera carcinoma in 18 cases (66.7%), followed by follicular carcinoma in six cases (22.2%), follicular variant papillifera carcinoma (two cases - 7.4%) and Hurthle cells carcinoma in one case (3.7%). In all these patients a total thyroidectomy was indicated in a period that varied between 15 and 30 days after the first intervention.

Of the 27 patients studied, in six of them (22.2%) the completion thyroidectomy was not carried out; four refused the second operation, and we lost their follow up; in one there was intense fibrosis in the surgical field and the last one had a micro-carcinoma. Therefore, 21 patients were submitted to thyroidectomy completion.

## RESULTS

**Figure 1:**
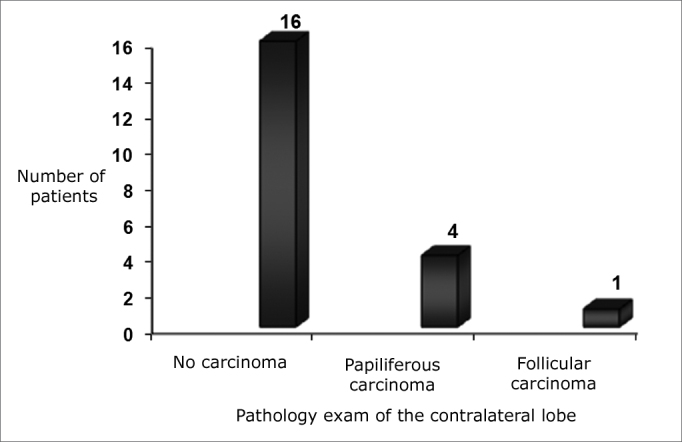
Number of patients in relation to the pathology exam of the surgical specimen from the second surgical intervention.

The pathology analysis of the contralateral lobe of the surgical specimen was negative for carcinoma in 16 (76.2%) and positive in the remaining five (23.8%) patients. In those whom the contralateral analysis was positive for carcinoma, the most frequently found histological pattern was the papillifera carcinoma in four, followed by follicular carcinoma in one (Figure 1).

In relation to post-operative complications, three patients (14.3%) had temporary dysphonia (one of them occurred in the first procedure and spontaneously reverted and the remaining two, after thyroidectomy complementation and reverted in 15 to 40 days). Another complication after thyroidectomy completion was hypoparathyroidism, which happened to two patients (9.5%), temporary in one - lasting for 35 days, and permanent in another. There was zero mortality.

## DISCUSSION

Most of the series studied have advocated total thyroidectomy for the surgical treatment of the thyroid differentiated carcinoma, simply because this procedure has advantages, such as: a greater sensitivity regarding the level of thyroglobulin to assess the recurrent disease, especially distant ones; ease of detection and ablation for distant metastasis by radiotherapy; prevent the possibility of residual thyroid differentiated carcinoma to turn anaplastic; and greater survival with a lower rate of recurrence^3^, ^4^, ^5^, ^6^, ^7^, ^8^, ^9^, ^10^, ^11^, ^12^, ^13^, ^14^.

Some series have shown that the possibility of the patient having residual tumor in the contralateral lobe is variable, from 21 to 62%, when total thyroidectomy is carried out for prophylaxis purposes^6^, ^9^, ^12^, ^15^. De Jong et al. Showed an incidence of 43% of contralateral lobe involvement in 100 patients; Kim et al.^16^ showed an incidence of 31% in 81 patients and Pacini et al.^17^, showed an incidence of 44% in 80 patients. These data match the ones in our series, which was of 23.8% (five in 21 patients).

Multicentricity in the primary lobe seems to be associated with the higher incidence of bilateral carcinoma, as it has been shown by other authors^12^, ^18^. Although this statement can be true, none of our patients had a multifocal primary lobe lesion. Since it was shown that the incidence of latent carcinoma could be high, the issue is what method would be safer in order to remove the remaining contralateral lobe without causing higher morbidity. Some authors believe the reoperation can be associated with a higher morbidity and thus have advocated ablation with radioactive iodine of the remaining thyroid tissue in lieu of completion thyroidectomy^19^. However, this procedure is challenged by many, because it has some disadvantages, such as the need for a high dose of radioactive iodine, long term development of parathyroid adenoma and teratogenicity in young women in their reproductive years^6^, ^20^, ^21^, ^22^. Thus, thyroidectomy completion has, once more, its importance and, if carried out by a competent team, carries low morbidity. Among the complications mentioned, dysphonia as a consequence of the recurrent laryngeal nerve involvement has been found in an incidence of 0 to 5%^4^, ^6^, ^7^, ^9^, ^10^, ^12^. However, in most cases, dysphonia is transitory, as it happened in our series. Although the incidence of this complication has been greater than the average expected in the literature - 14.3% in a study group of 27 patients -three patients recovered their voices in 15 to 40 days of postoperative, without showing any vocal fold sequelae, confirmed by laryngoscopy.

Another complication that has enjoyed some attention is hypoparathyroidism which, in our series, was present in 9.5% of the cases, matching the literature information, which varies from 0 to 16.8%^3^, ^4^, ^6^, ^7^, ^9^, ^12^. In one case, this complication was temporary, it reverted 35 days later with specific treatment, and such fact was shown by other authors^4^, ^6^, ^9^. De Jong et al. showed that hypoparathyroidism was present in 3% of the patients who were submitted to total completion thyroidectomy and used vitamin D and calcium for some months with clinical signs and symptoms reversion.

Despite the incidence below 3%, some authors, even those who favor thyroidectomy completion, have advocated surgical procedure interruption when there is too much fibrosis in the operating field, in order to avoid complications. Such fact happened to one of our patients, but we were unable to follow him up. Although some authors^9^ indicate that the thyroidectomy completion should be carried out between three and six months of the first surgical intervention in order to prevent complications, this is not what we advocate, in agreement with Kim et al.^16^, who indicate the second intervention as early as possible, as a means to reduce the chance of hematogenic and lymphatic spread of the residual tumor to the contralateral lobe of these patients. The cases studied were operated in the first 30 days of postoperative from the first intervention, and such fact also occurred in other series studied^4^, ^6^, ^10^, ^12^.

## CONCLUSIONS

Thyroidectomy completion is an important procedure in the treatment of a malignant thyroid tumor because of the high rate of contralateral involvement (23.8%). It is a procedure with zero mortality and very low complication rate, not bearing relevant risks.
